# Necrological

**Published:** 1881-09

**Authors:** 


### NECROLOGICAL.

"Death is the crown of life:
Were death denied, poor man would live in vain."

Prof. Byron F. Coy, D.D.S., died at his home in Baltimore, July 20, 1881, in his fifty-fifth year. He was a native of Maine, but lived and practised the last twenty-five years in the city where he came to his untimely end. He was a skillful operator, and had justly achieved a reputation second to none. He had been elected, and officiated with credit to himself, President of the American Dental Convention and of the Maryland and District of Columbia Dental Society; also for a number of years Professor in the Maryland Dental College. He was a contributor and subscriber to our journal, and his loss will not only be felt by his family (wife and three children) and many appreciative patients, but by the profession at large. B. M. W.

